# Supplementary material for: Anyon quantum dimensions from an arbitrary ground state wave function
Source: Nat Commun. 2024 Jun 15;15:5134. doi: 10.1038/s41467-024-47856-7 (PMC11180095; doi:10.1038/s41467-024-47856-7)
Supplement: Supplementary file 1 — Supplementary Information [file 41467_2024_47856_MOESM1_ESM.pdf]

Supplementary Information

Shang Liu[*]

Kavli Institute for Theoretical Physics, University of California, Santa Barbara, California 93106, USA

In this appendix, we apply our protocol to Kitaev's quantum double models [1]. We first review the models in Supplementary Note I and then implement our protocol in Supplementary Note II. In the last step of the protocol, we need some EE results in quantum double models. Details of the EE calculations are provided in Supplementary Note III.

Supplementary Note I: Quantum Double Models

Let us start by reviewing some definitions. There is a quantum double model for each finite group G (generally nonabelian). The model can live on an arbitrary lattice on an arbitrary orientable two-dimensional surface. The physical degrees of freedom, called spins, live on the edges, and the local Hilbert space of each spin is spanned by the orthonormal group element basis $\{|g\rangle \,|\, g \in G\}$. We need to choose a direction for each edge. Reversing the direction of a particular edge will be equivalent to the basis change $|z\rangle \mapsto |z^{-1}\rangle$ for the corresponding spin. Let v be a vertex, and f be an adjacent face, we define the local gauge transformations $A_v(g)$ and magnetic charge operators $B_{(v,f)}(h)$ as follows.

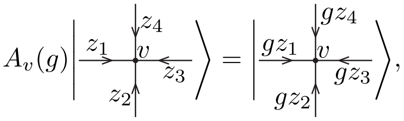

(1)

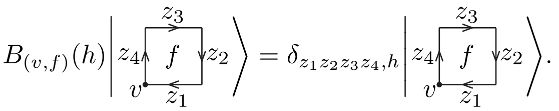

(2)

Here we use a tetravalent vertex and a square face as examples, and the generalizations should be straightforward. We further define two projectors:

$$A_v := |G|^{-1} \sum_{g\in G} A_v(g), \quad B_f := B_{(v,f)}(1). \tag{3}$$

Note that B_f does not depend on the choice of the adjacent vertex v. The quantum double Hamiltonian is then given by [1]

$$H_{\text{QD}} = -\sum_v A_v - \sum_f B_f. \tag{4}$$

The projectors in H_{QD} all commute with each other.

[*] sliu.phys@gmail.com

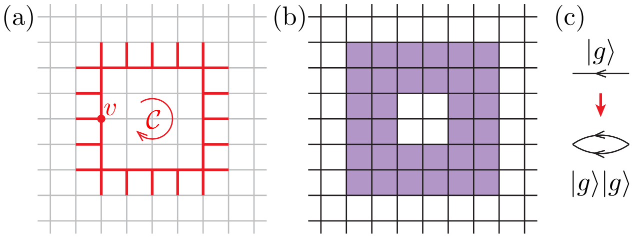

Supplementary Figure 1. Illustrations about the quantum doubled models. (a) Support of the operator $A_{\mathcal{C}}(g)$ for a loop $\mathcal{C}$ with base point v. (b) Bipartition into A (annulus) and B. (c) Duplication of a spin.

On a sphere, the model has a unique gapped ground state $|\Omega\rangle$ satisfying $A_v = B_f = 1$ for all vertices v and faces f. Let us introduce some useful properties of this state before implementing our protocol. More explicitly, we can write $|\Omega\rangle \propto (\prod_v A_v)(\bigotimes_e |1\rangle_e)$ where e runs over all edges. Using $[A_v(g), A_{v'}(h)] = 0$ for $v \neq v'$, and $A_v(g)A_v(h) = A_v(gh)$, one can check that

- $A_v(g)\,|\Omega\rangle = |\Omega\rangle$ for all v and g.

Given an oriented path along the edges from one vertex to another, when the relevant edge orientations are all consistent with the path direction, we define the holonomy of the path to be the product of all group elements on the path in the reversed order. For example, the holonomy of the following path from vertex u to v is given by $g_3 g_2 g_1$ (note the ordering).

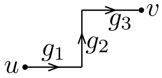

With this terminology, $B_f = 1$ means that the holonomy around any face is trivial. It follows that

- for the state $|\Omega\rangle$, the holonomy for any closed loop is trivial, and the holonomy between any two vertices does not depend on the choice of path.

Analogously, the property $A_v(g)\,|\Omega\rangle = |\Omega\rangle$ also has a generalization on loops. Given a loop $\mathcal{C}$ with a base point v, we can define operators $A_{\mathcal{C}}(g)$ whose support has the shape of a comb shown in Supplementary Fig. 1a. The action of $A_{\mathcal{C}}(g)$ is defined by

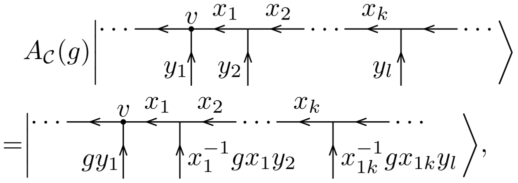

(5)

where $x_{1k} := x_1 x_2 \cdots x_k$. Let $\mathcal{L}_{\text{flat}}$ be the Hilbert subspace spanned by spin configurations satisfying $B_f = 1$ $\forall f$. $A_{\mathcal{C}}(g)$ preserves $\mathcal{L}_{\text{flat}}$. Moreover, $[A_{\mathcal{C}}(g), A_{v'}(h)] = 0$ for $v \neq v'$, and $A_{\mathcal{C}}(g)A_v(h) = A_v(h)A_{\mathcal{C}}(h^{-1}gh)$. Let $A_{\mathcal{C}} := |G|^{-1}\sum_{g\in G} A_{\mathcal{C}}(g)$. One can check $A_{\mathcal{C}}|\Omega\rangle = |\Omega\rangle$ with these commutation relations and the observation that acting $A_{\mathcal{C}}(g)$ on $\bigotimes_e |1\rangle_e$ is equivalent to acting several $A_v(g)$ operators. Using $A_{\mathcal{C}}(g)A_{\mathcal{C}}(h) = A_{\mathcal{C}}(gh)$, it follows that

- $A_{\mathcal{C}}(g)|\Omega\rangle = |\Omega\rangle$.

We will also call $A_{\mathcal{C}}(g)$ a gauge transformation operator.

Supplementary Note II: Applying the Protocol

With all these prerequisites, we are now ready to implement our protocol. Suppose a state $|\psi\rangle$ has no excitation with respect to H_{QD} in a large contractible region, and suppose this region has the form of a square lattice. It has been shown in Ref. 2 that the reduced density matrix of $|\psi\rangle$ deep inside this region has no dependence on the choice of $|\psi\rangle$. Hence, we will just take $|\psi\rangle$ to be the sphere ground state $|\Omega\rangle$. Consider a bipartition of the space like that in Supplementary Fig. 1b. Here, in order to draw the partition interface right on the edges, we imagine duplicating each edge into two, and require them to always be in the same group element state; see Supplementary Fig. 1c. This is just a simple trick inspired by Ref. 3 for getting a nice partition; each pair of spins obtained this way can still be regarded as a single spin unless the partition is being considered [4]. Still calling the state $|\Omega\rangle$, we can write

$$|\Omega\rangle = \sum_{\substack{g_A \text{ with trivial} \\ \text{holonomies}}} |g_A\rangle_A |\phi(g_A)\rangle_B\,, \tag{6}$$

where g_A's are spin configurations in the annulus region A, and $|\phi(g_A)\rangle_B$ are some set of states on B that are not necessarily normalized or orthogonal. In the summation above, we require g_A to have trivial holonomy around any loop in A, contractible or noncontractible.

We claim that for any two product states of group elements $|g_A\rangle$ and $|g'_A\rangle$ with trivial holonomies in A, and with the same group elements on the boundary ∂A, there exists a gauge transformation acting on A which transforms $|g_A\rangle$ to $|g'_A\rangle$. We can build such a transformation step by step: First choose the unique local gauge transformation $A_{v_0}(g_0)$ acting on the top left internal vertex such that $A_{v_0}(g_0)|g_A\rangle$ matches $|g'_A\rangle$ on the entire top left face. Then move rightward and choose the unique $A_{v_1}(g_1)$ acting on the next vertex v_1 such that $A_{v_1}(g_1)A_{v_0}(g_0)|g_A\rangle$ matches $|g'_A\rangle$ on the top left two faces. Continue this process until the top row of faces are all done, and start over from the left most vertex in the second row. When the face at the top left corner of the internal boundary of A is encountered, in order to not alter the spin configuration on ∂A, we need to utilize a loop operator $A_{\mathcal{C}}(g)$ to fix that face, where $\mathcal{C}$ coincides with the internal boundary. This kind of loop operators are no longer needed subsequently, and eventually g_A can be completely transformed into g'_A. Since the $A_v(g)$ and $A_{\mathcal{C}}(h)$ operators are all unitary and leave $|\Omega\rangle$ invariant, when g_A and g'_A are related by a gauge transformation, we have $|\phi(g_A)\rangle_B = {}_A\langle g_A|\Omega\rangle = {}_A\langle g'_A|\Omega\rangle = |\phi(g'_A)\rangle_B$. This means that $\phi(g_A)$ actually only depends on the spin configuration on ∂A. We can then write

$$|\Omega\rangle = \sum_{\substack{g_{\partial A} \text{ with trivial} \\ \text{holonomies}}} |\xi(g_{\partial A})\rangle_A |\phi(g_{\partial A})\rangle_B\,, \tag{7}$$

where $|\xi(g_{\partial A})\rangle$ is the sum of all holonomy free $|g_A\rangle$ such that $(g_A)|_{\partial A} = g_{\partial A}$.

The $|\phi(g_{\partial A})\rangle_B$ states are orthogonal to each other, because subsystem B contains a copy of the spin configuration on ∂A. We will now prove that they also have the same norm. Observe that any spin configuration $g_{\partial A}$ with trivial holonomies can be transformed into another $g'_{\partial A}$ using the local gauge transformations $A_v(g)$ overlapping with ∂A. Let U be that total gauge transformation operator. We can write $U = V_A V_B$ where V_A (V_B) is a unitary operator acting on A (B). From the definition of $|\xi(g_{\partial A})\rangle$, we can see $V_A|\xi(g_{\partial A})\rangle = |\xi(g'_{\partial A})\rangle$. It then follows from $U|\Omega\rangle = |\Omega\rangle$ that $V_B|\phi(g_{\partial A})\rangle = |\phi(g'_{\partial A})\rangle$. Hence $|\phi(g_{\partial A})\rangle$ and $|\phi(g'_{\partial A})\rangle$ indeed have the same norm.

The above analysis is inspired by Ref. 2. With these results established, we easily find

$$|\rho_A\rangle \propto \sum_{\substack{g_{\partial A} \text{ with trivial} \\ \text{holonomies}}} |\xi(g_{\partial A})\rangle |\xi(g_{\partial A})\rangle\,. \tag{8}$$

This state lives on the doubled system $A \cup A'$, i.e. two copies of the annulus in Supplementary Fig. 1b. Now imagine gluing the vertices in ∂A with the corresponding vertices in $\partial A'$, obtaining a torus. One can check that $|\rho_A\rangle$ is a ground state of the quantum double model defined on this torus. In particular, it is invariant under the actions of (new) local gauge transformations crossing the gluing interface. The quantum double model has multiple ground states on the torus. $|\rho_A\rangle$ is the special one characterized by trivial holonomy around the hole existing in each of the original annuli, and by $A_{\mathcal{C}}(g) = 1$ around the same hole. These actually imply trivial anyon flux through the hole, because any anyon flux would be detected by some loop operator that winds another anyon around it. We have thus successfully recovered the picture in Fig. 3c of the main text.

The next step is to obtain the desired mutual information. Although this has been done in the continuum, we did not find a lattice result meeting our need. We have thus performed an honest calculation, and it eventually works out magically. We refer interested readers to Supplementary Note III for the rather tedious details. The key technical trick is to use the holonomy basis introduced in Ref. 5: In the subspace with $B_f = 1$, labeling spin configurations by group elements on all edges contains a lot of redundancy, and we can instead label spin

configurations in each regions using independent holonomy variables. With this way of labeling basis vectors, the remaining calculation is more or less just brute-force.

We have found that the anyon sectors are labeled by a pair of variables (C, μ). Here, C labels a conjugacy class of G. Let $h_C \in C$ be a representative that is arbitrary but fixed once for all. μ labels an irreducible representation of $Z_C := \{g \in G | g h_C = h_C g\}$, the centralizer of h_C. The quantum dimensions are given by $d_{(C,\mu)} = |C| d_\mu$ where d_μ is the dimension of the representation μ. These are consistent with known results [1, 6, 7].

Supplementary Note III: EE Computations

In this appendix, we elaborate details about EE computations in quantum double models. We will utilize the technique of holonomy basis introduced in Ref. 5.

A. Holonomy Basis

Throughout this section, we restrict our attention to the Hilbert subspace $\mathcal{L}_{\text{flat}}$ spanned by spin configurations satisfying $B_f = 1$ for all f. Within this subspace, it is convenient to label spin configurations by independent holonomies: We choose some base point v_0, a path from v_0 to every other vertex, and a closed loop based at v_0 for each noncontractible cycle of the space. Then each spin configuration is uniquely determined by the holonomies along these paths and loops; one can solve the group element state on each edge from those holonomies.

As an example, let there be V number of vertices $v_0, w_1, w_2, \cdots, w_{V-1}$, and just one noncontractible circle. Denote by g_i the holonomy from v_0 to w_i along the chosen path, and by k the holonomy around the closed loop. We can write the basis vectors as $\{|g_i; k\rangle\}$. An operator $A_{w_i}(h)$ acts as

$$A_{w_i}(h)\,|g_j; k\rangle = |g_1, \cdots, hg_i, \cdots, g_{V-1}; k\rangle\,, \tag{9}$$

and $A_{v_0}(h)$ acts as

$$A_{v_0}(h)\,|g_j; k\rangle = |g_1 h^{-1}, \cdots, g_{V-1}h^{-1}; hkh^{-1}\rangle\,. \tag{10}$$

Note that despite the terminology "holonomy basis", we are still using the natural basis of tensor products of group elements. We just adopt a more convenient labeling of the basis vectors.

B. Warm-Up: EE of a Disk on a Sphere

As a warm-up, let us compute the EE for a disk region on a sphere. Denote this disk by A, and the complement (also a disk) by B. We define holonomy bases separately for the two regions, as shown in Supplementary Fig. 2. The base point in A (B) is v_A (v_B). Let there be L number of vertices $w_1, \cdots, w_L$ on the partition interface,

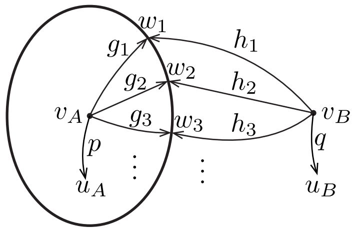

Supplementary Figure 2. Holonomies for the inside and outside of a disk.

we denote the holonomy from v_A (v_B) to w_i by g_i (h_i). There are also holonomies from v_A (v_B) to other internal vertices of A (B), but it turns out that these internal holonomies do not contribute to the EE. Therefore, for simplicity, we just retain one such internal vertex u_A (u_B) in A (B) and denote the corresponding holonomy by p (q).

We are interested in states with $B_f = 1$ for all faces f, which implies that the holonomy around any contractible loop is trivial. We thus have

$$g_1^{-1} h_1 = g_2^{-1} h_2 = \cdots = g_L^{-1} h_L =: a\,. \tag{11}$$

Take an arbitrary holonomy configuration $|g_i; h_i; p; q\rangle$. Imposing the above condition, we can write $h_i = g_i a$. The ground state $|\Omega\rangle$ can be obtained by applying the projectors A_v for all v to the state $|g_i; g_i a; p; q\rangle$. First consider the A_{w_i} operators. We have

$$\prod_{i=1}^{L} A_{w_i}\,|g_1, g_2, \cdots; g_1 a, g_2 a, \cdots; p; q\rangle \tag{12}$$

$$\propto \sum_{h_i} |h_1 g_1, h_2 g_2, \cdots; h_1 g_1 a, h_2 g_2 a, \cdots; p; q\rangle \tag{13}$$

$$= \sum_{g_i} |g_1, g_2, \cdots; g_1 a, g_2 a, \cdots; p; q\rangle\,. \tag{14}$$

Here, to obtain the last line, we first do a change of variable $h_i \mapsto h_i g_i^{-1}$ to absorb all g_i, and then rename the dummy variables h_i to g_i. We see that the net effect of the A_{w_i} operators is a summation over the g_i's. Similarly, applying A_{u_A} and A_{u_B} results in a summation over p and q. Next, applying A_{v_B}, we have

$$A_{v_B} \sum_{g_i, p, q} |g_i; g_i a; p; q\rangle \tag{15}$$

$$\propto \sum_{g_i, p, q, h_B} |g_i; g_i a h_B^{-1}; p; q h_B^{-1}\rangle \tag{16}$$

$$= \sum_{g_i, p, q, a} |g_i; g_i a; p; q\rangle\,. \tag{17}$$

We effectively get a summation over a. Finally, applying A_{v_A} to the above state has no effect. We have thus found

$$|\Omega\rangle \propto \sum_{g_i, p, q, a} |g_i; g_i a; p; q\rangle\,. \tag{18}$$

We now see that the internal vertices u_A and u_B just contribute a product state $(|G|^{-1}\sum_p |p\rangle)_A(|G|^{-1}\sum_q |q\rangle)_B$ which does not affect the EE. We will therefore ignore the p and q variables in the following, and simply write $|\Omega\rangle \propto \sum_{g_i,a} |g_i; g_i a\rangle$.

Taking a partial trace over subsystem A, we obtain

$$\rho_B \propto \sum_{g_i,a,b} |g_i a\rangle \langle g_i b| \,. \tag{19}$$

For later convenience, we do a change of variable: $a \mapsto g_1^{-1}a$, $b \mapsto g_1^{-1}b$, $g_{i>1} \mapsto g_{i>1}g_1$. It follows that

$$\rho_B \propto \sum_{g_{i>1},a,b} |a, g_2 a, g_3 a, \cdots\rangle \langle b, g_2 b, g_3 b, \cdots| \,. \tag{20}$$

We denote by $\tilde{\rho}_B$ the unnormalized density matrix on the right-hand side.

To compute the Renyi EE, we need to evaluate $\mathrm{Tr}(\tilde{\rho}_B^n)$. We add a superscript $\mu = 1, 2, \cdots, n$ to the dummy variales in the μ-th copy of $\tilde{\rho}_B$, and it is not hard to see that

$$b^\mu = a^{\mu+1}, \quad g_{i>1}^\mu = g_{i>1}^{\mu+1} =: g_{i>1}. \tag{21}$$

We are left with

$$\mathrm{Tr}(\tilde{\rho}_B^n) = \sum_{a^\mu, g_{i>1}} 1 = |G|^{n+L-1}. \tag{22}$$

The Renyi EE is then

$$S_A^{(n)} = S_B^{(n)} = \frac{1}{1-n} \log \left[\frac{\mathrm{Tr}(\tilde{\rho}_B^n)}{\mathrm{Tr}(\tilde{\rho}_B)^n} \right] = (L-1)\log|G|. \tag{23}$$

We can identify the $L \log|G|$ term as the "area-law" term proportional to the length of the partition interface, and identify $-\log|G|$ as the universal topological EE [3, 8]. It follows that $\mathcal{D} = |G|$.

Our calculation above essentially follows that in Ref. 5. The same result is also obtained in Ref. 2 with different approaches.

C. EEs on a Torus

Let us now tackle the real problem. Recall that we need to compute the mutual information between two disjoint annulus regions of a torus. To the end, we need to compute the EEs both of a single annulus, and of two annuli. Let us then imagine partitioning the torus into $2N$ number of annuli, denoted by $T_1, T_2, \cdots, T_{2N}$. We will be interested only in the cases $N = 1$ and $N = 2$, but it will be convenient to keep a unified notation.

We define holonomy bases separately for the $2N$ regions. In Supplementary Fig. 3, we show our holonomy variables for the case of $N = 2$, and generalizations to other values of N should be clear. We denote by v_I with $I \in \{1, 2, \cdots, 2N\}$ the base point in the region T_I. From

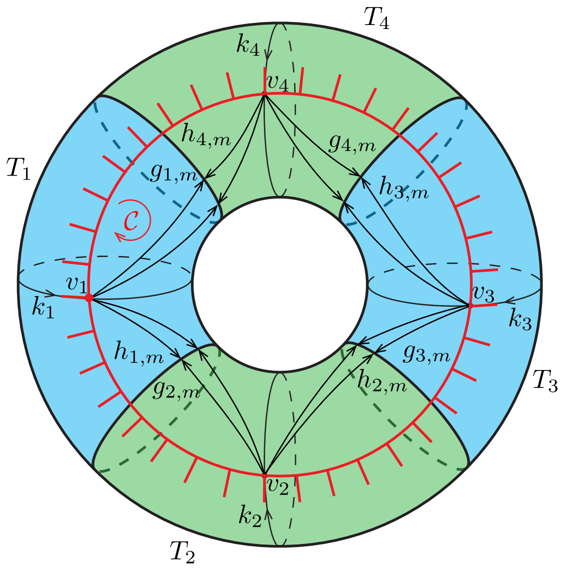

Supplementary Figure 3. Holonomy variables for a tetrapartite torus, and the support (red comb) of the loop operators $A_{\mathcal{C}}(g)$.

each v_I, there are holonomies $g_{I,m}$ and $h_{I,m}$ to the interfaces with T_{I-1} and T_{I+1}, respectively. We assume the numbers of vertices on all the interface circles are the same, denoted by L, just for the simplicity of notations. There is also a holonomy k_I around a noncontractible loop in each region. As in the previous example, internal vertices will not contribute to the EE, so we have simply omitted them. We draw a loop $\mathcal{C}$ that is based at v_1 and passes through all the other v_I. The comb-like support of $A_{\mathcal{C}}(g)$ operators associated with the loop is also plotted. As we have shown in the main text, the special ground state $|\Omega\rangle$ we consider has trivial holonomy around $\mathcal{C}$, and satisfies $A_{\mathcal{C}}|\Omega\rangle = |\Omega\rangle$.

Let us start from some holonomy configuration $|g_{I,m}; h_{I,m}; k_I\rangle$. We would like to impose $B_f = 1$ for all faces f, or equivalently, every contractible loop has trivial holonomy. Then $g_{I+1,m}^{-1}h_{I,m}$ should be independent of m, and we will denote this quantity by a_I. In other words, $h_{I,m} = g_{I+1,m}a_I$. The $B_f = 1$ conditions also imply $k_I a_I^{-1} k_{I+1}^{-1} a_I = 1$. We can thus write

$$k_{I+1} = a_I a_{I-1} \cdots a_1 k_1 a_1^{-1} \cdots a_{I-1}^{-1} a_I^{-1}. \tag{24}$$

The condition of trivial holonomy around $\mathcal{C}$ implies that

$$a_{2N} a_{2N-1} \cdots a_1 = 1. \tag{25}$$

To obtain the desired ground state $|\Omega\rangle$, we shall apply the projectors $A_{\mathcal{C}}$, $A_{I,m}$, and A_{v_I} to $|g_{I,m}; h_{I,m}; k_I\rangle$, where $A_{I,m}$ is the local gauge invariance projector acting on the m-th vertex of the $T_I T_{I+1}$ interface (affecting $h_{I,m}$ and $g_{I+1,m}$).

We can choose the paths for $g_{I,m}$ and $h_{I,m}$ to have no overlap with the support of $A_{\mathcal{C}}(z)$ operators. The same holds for paths from v_I to other internal vertices, which

we have omitted. As a result, $A_\mathcal{C}(z)$ will only affect k_I, and its action is more explicitly given by

$$k_I \mapsto (a_{I-1}\cdots a_1 z a_1^{-1}\cdots a_{I-1}^{-1})k_I \tag{26}$$
$$= a_{I-1}\cdots a_1 (zk_1) a_1^{-1}\cdots a_{I-1}^{-1}. \tag{27}$$

Hence, after the action of $A_\mathcal{C}$, the state $|g_{I,m}; h_{I,m}; k_I\rangle$ becomes

$$\sum_{k_1} |g_{I,m}; g_{I+1,m}a_I; a_{I-1}\cdots a_1 k_1 a_1^{-1}\cdots a_{I-1}^{-1}\rangle\,, \tag{28}$$

up to a normalization factor. The actions of $A_{I,m}$ effectively induce summations over $g_{I,m}$ for the above expression. Finally, applying A_{v_I}, we obtain

$$|\Omega\rangle \propto \sum_{k_1,\ g_{I,m},\ h_I} |g_{I,m}h_I^{-1}; g_{I+1,m}a_I h_I^{-1}; h_I a_{I-1}a_{I-2}\cdots a_1 k_1 a_1^{-1}\cdots a_{I-2}^{-1}a_{I-1}^{-1}h_I^{-1}\rangle \tag{29}$$
$$= \sum_{k_1,\ g_{I,m},\ h_I} |g_{I,m}; g_{I+1,m}(h_{I+1}a_I h_I^{-1}); (h_I a_{I-1}h_{I-1}^{-1})(h_{I-1}a_{I-2}h_{I-2}^{-1})\cdots(h_2 a_1 h_1^{-1})k_1\cdots\rangle\,, \tag{30}$$

where the second line is obtained by a change of variables: $g_{I,m}\mapsto g_{I,m}h_I$ and $k_1 \mapsto h_1^{-1}k_1 h_1$. Now observe that the following map within $G^{\times 2N}$ is one-to-one:

$$(h_{2N}, h_{2N-1}, \cdots, h_1) \mapsto (h_{2N}a_{2N-1}h_{2N-1}^{-1}, h_{2N-1}a_{2N-2}h_{2N-2}^{-1}, \cdots, h_2 a_1 h_1^{-1}, h_1). \tag{31}$$

Therefore, summing over the variables on the left is equivalent to summing over those on the right. Using this fact, together with Supplementary Eq. 25 that implies $(h_1 a_{2N} h_{2N}^{-1})(h_{2N}a_{2N-1}h_{2N-1}^{-1})\cdots(h_2 a_1 h_1^{-1}) = 1$, we obtain

$$|\Omega\rangle \propto \sum_{k_1,\ a_I,\ g_{I,m}} \delta(a_{2N}a_{2N-1}\cdots a_1, 1)\, |g_{I,m}; g_{I+1,m}a_I; a_{I-1}\cdots a_1 k_1 a_1^{-1}\cdots a_{I-1}^{-1}\rangle\,, \tag{32}$$

where $\delta(\cdot,\cdot)$ is the Kronecker delta function.

Partial tracing $|\Omega\rangle\langle\Omega|$ over $T_1, T_3, T_5, \cdots, T_{2N-1}$, and after a simple change of variables, we find the following reduced density matrix.

$$\rho \propto \sum_{k,\ g_{I,m},\ a_I,\ b_I} \delta(a_{2N}\cdots a_1, 1)\delta(b_{2N}\cdots b_1, 1)\delta(a_{2K-2}\cdots a_1 k a_1^{-1}\cdots a_{2K-2}^{-1}, b_{2K-2}\cdots b_1 k b_1^{-1}\cdots b_{2K-2}^{-1})|_{2\le K\le N}$$
$$|g_{2K,m}a_{2K-1}^{-1}; g_{2K+1,m}a_{2K}; a_{2K-1}\cdots a_1 k a_1^{-1}\cdots a_{2K-1}^{-1}\rangle\langle g_{2K,m}b_{2K-1}^{-1}; g_{2K+1,m}b_{2K}; b_{2K-1}\cdots b_1 k b_1^{-1}\cdots b_{2K-1}^{-1}| =: \tilde{\rho}. \tag{33}$$

We have introduced a new index K which takes values from $\{1, 2, \cdots, N\}$ unless otherwise indicated (like for the third δ above). Now apply the following change of variables:

$$a_{2K-1}\mapsto a_{2K-1}g_{2K,1}, \quad b_{2K-1}\mapsto b_{2K-1}g_{2K,1}, \quad g_{2K,m>1}\mapsto g_{2K,m}g_{2K,1}, \tag{34}$$
$$a_{2K}\mapsto g_{2K+1,1}^{-1}a_{2K}, \quad b_{2K}\mapsto g_{2K+1,1}^{-1}b_{2K}, \quad g_{2K+1,m>1}\mapsto g_{2K+1,m}g_{2K+1,1}. \tag{35}$$

We obtain

$$\tilde{\rho} = \sum_{k,\ g_{I,m},\ a_I,\ b_I} \delta(g_{1,1}^{-1}a_{2N}a_{2N-1}g_{2N,1}g_{2N-1,1}^{-1}a_{2N-2}\cdots a_1 g_{2,1}, 1)\delta(g_{1,1}^{-1}b_{2N}b_{2N-1}g_{2N,1}g_{2N-1,1}^{-1}b_{2N-2}\cdots b_1 g_{2,1}, 1)$$
$$\delta(g_{2K-1,1}^{-1}a_{2K-2}a_{2K-3}g_{2K-2,1}\cdots a_1 g_{2,1}k\cdots, g_{2K-1,1}^{-1}b_{2K-2}b_{2K-3}g_{2K-2,1}\cdots b_1 g_{2,1}k\cdots)|_{K>1}$$
$$|(1, g_{2K,m>1})a_{2K-1}^{-1}; (1, g_{2K+1,m>1})a_{2K}; a_{2K-1}g_{2K,1}g_{2K-1,1}^{-1}a_{2K-2}a_{2K-3}\cdots a_1 g_{2,1}k\cdots\rangle$$
$$\langle(1, g_{2K,m>1})b_{2K-1}^{-1}; (1, g_{2K+1,m>1})b_{2K}; b_{2K-1}g_{2K,1}g_{2K-1,1}^{-1}b_{2K-2}b_{2K-3}\cdots b_1 g_{2,1}k\cdots|\,, \tag{36}$$

an incredibly complicated expression! This step is similar to the one we took in the warm-up example before evaluating $\mathrm{Tr}(\tilde{\rho}_B^n)$. Here and below, we often write terms of the form xkx^{-1} as $xk\cdots$ when x has a long expression. In the first entry of the ket, the notation $(1, g_{2K,m>1})$ represents a list of group elements indexed by m. $m=1$ corresponds to the identity 1, and each $m>1$ corresponds to $g_{2K,m>1}$. These elements are then all multiplied by a_{2K-1}^{-1} from the right. Other similar entries can be interpreted analogously. Now we are ready to compute $\mathrm{Tr}(\tilde{\rho}^n)$. We again add a superscript μ to the dummy variables of the μ-th copy of $\tilde{\rho}$. We see that $b_I^\mu = a_I^{\mu+1}$. The summations over b_I^μ can then

be dropped. In addition, $g^{\mu}_{I,m>1} = g^{\mu+1}_{I,m>1}$. We can now do the summations over $g^{\mu}_{I,m>1}$ and get a factor $|G|^{2N(L-1)}$. We rename $g^{\mu}_{I,1}$ as g^{μ}_{I}, and we are left with

$$\begin{aligned}\mathrm{Tr}(\tilde{\rho}^n) = &\sum_{k^\mu, g^\mu_I, a^\mu_I} |G|^{2N(L-1)} \\ &\delta[(g^\mu_1)^{-1} a^\mu_{2N} a^\mu_{2N-1} g^\mu_{2N} (g^\mu_{2N-1})^{-1} a^\mu_{2N-2} \cdots a^\mu_1 g^\mu_2, 1] \delta[(g^\mu_1)^{-1} a^{\mu+1}_{2N} a^{\mu+1}_{2N-1} g^\mu_{2N} (g^\mu_{2N-1})^{-1} a^{\mu+1}_{2N-2} \cdots a^{\mu+1}_1 g^\mu_2, 1] \\ &\delta[(g^\mu_{2K-1})^{-1} a^\mu_{2K-2} a^\mu_{2K-3} g^\mu_{2K-2} \cdots a^\mu_1 g^\mu_2 k^\mu \cdots, (g^\mu_{2K-1})^{-1} a^{\mu+1}_{2K-2} a^{\mu+1}_{2K-3} g^\mu_{2K-2} \cdots a^{\mu+1}_1 g^\mu_2 k^\mu \cdots]|_{K>1} \\ &\delta[a^{\mu+1}_{2K-1} g^\mu_{2K} (g^\mu_{2K-1})^{-1} a^{\mu+1}_{2K-2} a^{\mu+1}_{2K-3} \cdots a^{\mu+1}_1 g^\mu_2 k^\mu \cdots, a^{\mu+1}_{2K-1} g^{\mu+1}_{2K} (g^{\mu+1}_{2K-1})^{-1} a^{\mu+1}_{2K-2} a^{\mu+1}_{2K-3} \cdots a^{\mu+1}_1 g^{\mu+1}_2 k^\mu \cdots]\end{aligned} \tag{37}$$

It seems too hard to proceed with this expression for a general N, so in the following, we will restrict to $N = 1$ and $N = 2$ which are the cases we need.

1. N=1

When $N = 1$, K can only be 1, and we find

$$\begin{aligned}\mathrm{Tr}(\tilde{\rho}^n) = &\sum_{k^\mu, g^\mu_I, a^\mu_I} |G|^{2L-2} \\ &\delta[(g^\mu_1)^{-1} a^\mu_2 a^\mu_1 g^\mu_2, 1] \delta[(g^\mu_1)^{-1} a^{\mu+1}_2 a^{\mu+1}_1 g^\mu_2, 1] \\ &\delta(a^{\mu+1}_1 g^\mu_2 k^\mu \cdots, a^{\mu+1}_1 g^{\mu+1}_2 k^{\mu+1} \cdots).\end{aligned} \tag{38}$$

Notice that in the last delta function, $a^{\mu+1}_1$ appears in both entries and thus can be removed. We can make a change of variables: $a^\mu_1 \mapsto (a^\mu_2)^{-1} a^\mu_1$, and $k^\mu \mapsto (g^\mu_2)^{-1} k^\mu g^\mu_2$. The a^μ_2 summation can now be done and gives $|G|^n$. The third δ function becomes $\delta(k^\mu, k^{\mu+1})$. The summation over k^μ therefore gives a factor of $|G|$. We are left with

$$\begin{aligned}\mathrm{Tr}(\tilde{\rho}^n) = &\sum_{g^\mu_I, a^\mu_1} |G|^{2L-1+n} \\ &\delta[(g^\mu_1)^{-1} a^\mu_1 g^\mu_2, 1] \delta[(g^\mu_1)^{-1} a^{\mu+1}_1 g^\mu_2, 1].\end{aligned} \tag{39}$$

We can do the summation over a^μ_1, and get

$$\mathrm{Tr}(\tilde{\rho}^n) = \sum_{g^\mu_I} |G|^{2L-1+n} \delta[g^\mu_1 (g^\mu_2)^{-1}, g^{\mu+1}_1 (g^{\mu+1}_2)^{-1}] \tag{40}$$

$$= \sum_{g^\mu_I} |G|^{2L-1+n} \delta[g^\mu_1, g^{\mu+1}_1] = |G|^{2L+2n}. \tag{41}$$

The Renyi EE can now be computed:

$$S^{(n)}_{T_2} = S^{(n)}_{T_1} = \frac{1}{1-n} \log \left[\frac{\mathrm{Tr}(\tilde{\rho}^n)}{(\mathrm{Tr}\,\tilde{\rho})^n} \right] = 2L \log |G|. \tag{42}$$

Recall that we have assumed the two interface circles between T_1 and T_2 to have the same length L. In general the $2L$ factor above should be replaced by the total interface length. We see that the Renyi EE contains only an area-law term, so the topological EE in this case actually vanishes. This is consistent with the field-theoretic result.

2. N=2

Plugging $N = 2$ and $K = 1, 2$ into Supplementary Eq.37, we find

$$\begin{aligned}\mathrm{Tr}(\tilde{\rho}^n) = &\sum_{k^\mu, g^\mu_I, a^\mu_I} |G|^{4(L-1)} \\ &\delta[(g^\mu_1)^{-1} a^\mu_4 a^\mu_3 g^\mu_4 (g^\mu_3)^{-1} a^\mu_2 a^\mu_1 g^\mu_2, 1] \\ &\delta[(g^\mu_1)^{-1} a^{\mu+1}_4 a^{\mu+1}_3 \cdots a^{\mu+1}_1 g^\mu_2, 1] \\ &\delta[(g^\mu_3)^{-1} a^\mu_2 a^\mu_1 g^\mu_2 k^\mu \cdots, (g^\mu_3)^{-1} a^{\mu+1}_2 a^{\mu+1}_1 g^\mu_2 k^\mu \cdots] \\ &\delta[a^{\mu+1}_1 g^\mu_2 k^\mu \cdots, a^{\mu+1}_1 g^{\mu+1}_2 k^\mu \cdots] \\ &\delta[a^{\mu+1}_3 g^\mu_4 (g^\mu_3)^{-1} a^{\mu+1}_2 a^{\mu+1}_1 g^\mu_2 k^\mu \cdots, \\ &\quad a^{\mu+1}_3 g^{\mu+1}_4 (g^{\mu+1}_3)^{-1} a^{\mu+1}_2 a^{\mu+1}_1 g^{\mu+1}_2 k^\mu \cdots].\end{aligned} \tag{43}$$

The last two delta functions here come from the last delta function of Supplementary Eq. 37 with $K = 1, 2$, respectively. Utilizing the first two delta functions, the last one can be simplified to

$$\delta[(a^{\mu+1}_4)^{-1} g^\mu_1 k^\mu \cdots, (a^{\mu+1}_4)^{-1} g^{\mu+1}_1 k^{\mu+1} \cdots]. \tag{44}$$

Further removing some obvious redundancies in the delta functions, we are left with

$$\begin{aligned}\mathrm{Tr}(\tilde{\rho}^n) = &\sum_{k^\mu, g^\mu_I, a^\mu_I} |G|^{4(L-1)} \\ &\delta[(g^\mu_1)^{-1} a^\mu_4 a^\mu_3 g^\mu_4 (g^\mu_3)^{-1} a^\mu_2 a^\mu_1 g^\mu_2, 1] \\ &\delta[(g^\mu_1)^{-1} a^{\mu+1}_4 a^{\mu+1}_3 g^\mu_4 (g^\mu_3)^{-1} a^{\mu+1}_2 a^{\mu+1}_1 g^\mu_2, 1] \\ &\delta[a^\mu_2 a^\mu_1 g^\mu_2 k^\mu \cdots, a^{\mu+1}_2 a^{\mu+1}_1 g^\mu_2 k^\mu \cdots] \\ &\delta[g^\mu_2 k^\mu (g^\mu_2)^{-1}, g^{\mu+1}_2 k^{\mu+1} (g^{\mu+1}_2)^{-1}] \\ &\delta[g^\mu_1 k^\mu (g^\mu_1)^{-1}, g^{\mu+1}_1 k^{\mu+1} (g^{\mu+1}_1)^{-1}].\end{aligned} \tag{45}$$

Now we do a change of variables: $g^\mu_4 \mapsto g^\mu_4 g^\mu_3$, $a^\mu_3 \mapsto (a^\mu_4)^{-1} a^\mu_3$, $a^\mu_1 \mapsto (a^\mu_2)^{-1} a^\mu_1$. The summations over g^μ_3, a^μ_4 and a^μ_2 can now be done and give $|G|^{3n}$. We also do the summation over g^μ_4 which reduces the first two delta

functions into a single one. We get

$$\begin{aligned}\mathrm{Tr}(\tilde{\rho}^n) = |G|^{4(L-1)+3n} \sum_{k^\mu, g_1^\mu, g_2^\mu, a_1^\mu, a_3^\mu} & \delta[a_1^\mu g_2^\mu (g_1^\mu)^{-1} a_3^\mu, a_1^{\mu+1} g_2^\mu (g_1^\mu)^{-1} a_3^{\mu+1}] \\ & \delta[a_1^\mu g_2^\mu k^\mu \cdots, a_1^{\mu+1} g_2^\mu k^\mu \cdots] \\ & \delta[g_2^\mu k^\mu (g_2^\mu)^{-1}, g_2^{\mu+1} k^{\mu+1} (g_2^{\mu+1})^{-1}] \\ & \delta[g_1^\mu k^\mu (g_1^\mu)^{-1}, g_1^{\mu+1} k^{\mu+1} (g_1^{\mu+1})^{-1}]. \end{aligned} \quad (46)$$

One more change of variables: $k^\mu \mapsto (g_2^\mu)^{-1} k^\mu g_2^\mu$, and $g_1^\mu \mapsto g_1^\mu g_2^\mu$. The summation over g_2^μ can be done to give $|G|^n$. Renaming g_1^μ as g^μ, we get

$$\begin{aligned}\mathrm{Tr}(\tilde{\rho}^n) = |G|^{4(L-1)+4n} \sum_{k^\mu, g^\mu, a_1^\mu, a_3^\mu} & \delta[a_1^\mu (g^\mu)^{-1} a_3^\mu, a_1^{\mu+1} (g^\mu)^{-1} a_3^{\mu+1}] \\ & \delta[a_1^\mu k^\mu (a_1^\mu)^{-1}, a_1^{\mu+1} k^\mu (a_1^{\mu+1})^{-1}] \delta[k^\mu, k^{\mu+1}] \\ & \delta[g^\mu k^\mu (g^\mu)^{-1}, g^{\mu+1} k^{\mu+1} (g^{\mu+1})^{-1}] \quad (47) \\ = |G|^{4(L-1)+4n} \sum_{k, g^\mu, a_1^\mu, a_3^\mu} & \delta[a_1^\mu (g^\mu)^{-1} a_3^\mu, a_1^{\mu+1} (g^\mu)^{-1} a_3^{\mu+1}] \\ & \delta[a_1^\mu k (a_1^\mu)^{-1}, a_1^{\mu+1} k (a_1^{\mu+1})^{-1}] \\ & \delta[g^\mu k (g^\mu)^{-1}, g^{\mu+1} k (g^{\mu+1})^{-1}]. \quad (48) \end{aligned}$$

The last delta function is equivalent to

$$\begin{aligned} & \delta[g^1 k (g^1)^{-1}, g^\mu k (g^\mu)^{-1}]|_{\mu>1} \\ = & \ \delta[k, (g^1)^{-1} g^\mu k (g^\mu)^{-1} g^1]|_{\mu>1}. \end{aligned} \quad (49)$$

We do a change of variables: $g^{\mu>1} \mapsto g^1 g^{\mu>1}$, and $a_3^\mu \mapsto g^1 a_3^\mu$. The summation over g^1 can now be done and gives $|G|$. We get

$$\begin{aligned}\mathrm{Tr}(\tilde{\rho}^n) = |G|^{4L-3+4n} \sum_{k, g^{\mu>1}, a_1^\mu, a_3^\mu} & \delta(a_1^1 a_3^1, a_1^2 a_3^2) \delta[a_1^\mu (g^\mu)^{-1} a_3^\mu, a_1^{\mu+1} (g^\mu)^{-1} a_3^{\mu+1}]|_{\mu>1} \\ & \delta[a_1^\mu k (a_1^\mu)^{-1}, a_1^{\mu+1} k (a_1^{\mu+1})^{-1}] \\ & \delta[k, g^\mu k (g^\mu)^{-1}]|_{\mu>1}. \end{aligned} \quad (50)$$

Notice that although the second last delta function applies to all μ, only $n-1$ values of μ give independent constraints. Hence, we can restrict that delta function to $2 \le \mu \le n$. We further rewrite

$$\begin{aligned}\mathrm{Tr}(\tilde{\rho}^n) = |G|^{4L-3+4n} \sum_{k, g^{\mu>1}, a_1^\mu, a_3^\mu} & \delta[(a_1^2)^{-1} a_1^1 a_3^1 (a_3^2)^{-1}, 1] \\ & \delta[(g^\mu)^{-1}, (a_1^\mu)^{-1} a_1^{\mu+1} (g^\mu)^{-1} a_3^{\mu+1} (a_3^\mu)^{-1}]|_{\mu>1} \\ & \delta[k, (a_1^\mu)^{-1} a_1^{\mu+1} k (a_1^{\mu+1})^{-1} (a_1^\mu)]|_{\mu>1} \\ & \delta[k, g^\mu k (g^\mu)^{-1}]|_{\mu>1}. \end{aligned} \quad (51)$$

Now define some variables:

$$\begin{aligned} & (x^1, x^2, x^3, \cdots, x^n) = \\ & [a_1^2, (a_1^2)^{-1} a_1^3, (a_1^3)^{-1} a_1^4, \cdots, (a_1^n)^{-1} a_1^1], \end{aligned} \quad (52)$$

$$\begin{aligned} & (y^1, y^2, y^3, \cdots, y^n) = \\ & [a_3^2, a_3^3 (a_3^2)^{-1}, a_3^4 (a_3^3)^{-1}, \cdots, a_3^1 (a_3^n)^{-1}]. \end{aligned} \quad (53)$$

The maps from a_1^μ, a_3^μ to x^μ, y^μ are one-to-one, so we can change the summation variables to x^μ and y^μ. The summations over x^1 and y^1 can then be done, giving $|G|^2$. We get

$$\begin{aligned}\mathrm{Tr}(\tilde{\rho}^n) = |G|^{4L-1+4n} \sum_{k, g^{\mu>1}, x^{\mu>1}, y^{\mu>1}} & \delta(x^2 x^3 \cdots x^n y^n \cdots y^3 y^2, 1) \\ & \delta[(g^\mu)^{-1}, x^\mu (g^\mu)^{-1} y^\mu]|_{\mu>1} \\ & \delta[k, x^\mu k (x^\mu)^{-1}]|_{\mu>1} \delta[k, g^\mu k (g^\mu)^{-1}]|_{\mu>1}. \end{aligned} \quad (54)$$

Using the second delta function, we find $(y^\mu)^{-1} = g^\mu x^\mu (g^\mu)^{-1}$. We then have

$$\begin{aligned}\mathrm{Tr}(\tilde{\rho}^n) = |G|^{4L-1+4n} \sum_{k, g^{\mu>1}, x^{\mu>1}} & \delta[x^2 x^3 \cdots x^n, g^2 x^2 (g^2)^{-1} g^3 x^3 (g^3)^{-1} \cdots g^n x^n (g^n)^{-1}] \\ & \delta[k, x^\mu k (x^\mu)^{-1}]|_{\mu>1} \delta[k, g^\mu k (g^\mu)^{-1}]|_{\mu>1}. \end{aligned} \quad (55)$$

The last two delta functions imply that x^μ and g^μ both belong to the centralizer of k, namely $Z(k) := \{g \in G | gk = kg\}$. We obtain the result

$$\begin{aligned}\mathrm{Tr}(\tilde{\rho}^n) = |G|^{4L-1+4n} \sum_{k \in G} \sum_{g^{\mu>1}, x^{\mu>1} \in Z(k)} \\ \delta[x^2 x^3 \cdots x^n, g^2 x^2 (g^2)^{-1} g^3 x^3 (g^3)^{-1} \cdots g^n x^n (g^n)^{-1}]. \end{aligned} \quad (56)$$

We can no longer simplify this expression just by changing variables, but it can be computed using nonabelian Fourier transforms. We summarize the key step as the following lemma.

Lemma 1. *Let H be a finite group, z_i and h_i with $i = 1, 2, 3, \cdots, r$ be group elements. Then,*

$$\begin{aligned} & \sum_{h_i, z_i \in H} \delta[z_1 z_2 \cdots z_r, (h_1 z_1 h_1^{-1})(h_2 z_2 h_2^{-1}) \cdots (h_r z_r h_r^{-1})] \\ & = |H|^{2r-1} \sum_{\mu \in \mathrm{Irrep}(H)} d_\mu^{2-2r}, \end{aligned} \quad (57)$$

where $\mathrm{Irrep}(H)$ *is the set of irreducible representations of* H*, and* d_μ *is the dimension of the representation* μ*.*

Proof. Denote by $L^2(H)$ the Hilbert space generated by the orthonormal basis $\{|h\rangle \, | h \in H\}$. Another orthonormal basis of $L^2(H)$ consists of the following states.

$$|\mu; a, b\rangle = \sqrt{\frac{d_\mu}{|H|}} \sum_{h \in H} \bar{D}^\mu_{ab}(h) \, |h\rangle \, . \quad (58)$$

Here, $\mu \in \mathrm{Irrep}(H)$, $a, b \in \{1, 2, \cdots, d_\mu\}$, $D^\mu(h)$ is the matrix for h in the representation μ, and $\bar{D}^\mu(h)$ is the complex conjugate of $D^\mu(h)$. The orthonormality of this new basis lies in Schur's orthogonality relation:

$$\sum_{h\in H} \bar{D}^\mu_{ab}(h) D^\nu_{cd}(h) = \frac{|H|}{d_\mu}\delta_{\mu\nu}\delta_{ac}\delta_{bd}. \tag{59}$$

The inverse map is given by

$$|h\rangle = \sum_{\mu\in\mathrm{Irrep}(H)} \sum_{a,b=1}^{d_\mu} \sqrt{\frac{d_\mu}{|H|}} D^\mu_{ab}(h)\, |\mu; a, b\rangle \,. \tag{60}$$

This is called the nonabelian Fourier transform.

Denote by $\mathcal{I}$ the summation in the statement of the lemma. We can rewrite the delta function there as an inner product in $L^2(H)$:

$$\mathcal{I} = \sum_{h_i, z_i \in H} \langle (h_1 z_1 h_1^{-1}) \cdots (h_r z_r h_r^{-1}) | z_1 \cdots z_r \rangle \,. \tag{61}$$

Applying nonabelian Fourier transforms,

$$\mathcal{I} = \sum_{h_i, z_i} \sum_{\mu} \sum_{a,b=1}^{d_\mu} \frac{d_\mu}{|H|} \bar{D}^\mu_{ab}(h_1 z_1 h_1^{-1} \cdots) D^\mu_{ab}(z_1 z_2 \cdots z_r). \tag{62}$$

It is now useful to introduce a diagrammatic language:

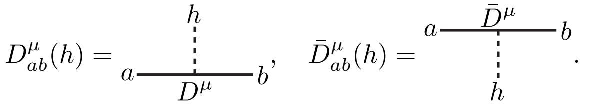

(63)

The orthogonality relation then looks like

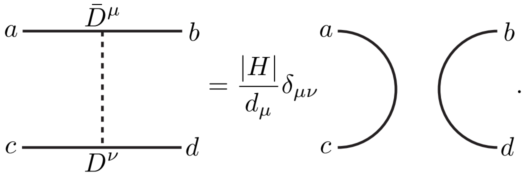

(64)

Using this diagrammatic language, and the fact that D^μ matrices satisfy the group multiplication laws, we compute the summations over z_i as

$$\sum_{z_i} \bar{D}^\mu_{ab}(h_1 z_1 h_1^{-1} \cdots) D^\mu_{ab}(z_1 z_2 \cdots z_r)$$

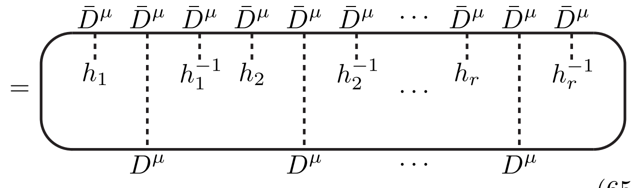

(65)

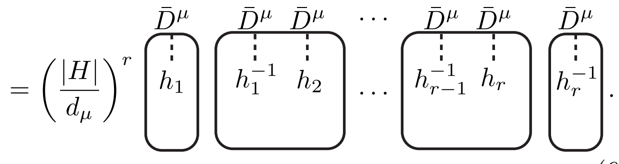

(66)

Since all the representations are unitary, we have $\bar{D}^\mu_{ab}(h^{-1}) = D^\mu_{ba}(h)$. This implies, for example,

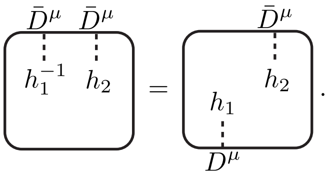

(67)

We can now do the summations over h_i, and find

$$\sum_{h_i}\sum_{z_i} \bar{D}^\mu_{ab}(h_1 z_1 h_1^{-1} \cdots) D^\mu_{ab}(z_1 z_2 \cdots z_r)$$

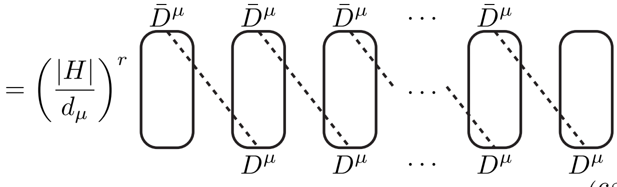

(68)

$= \left(\frac{|H|}{d_\mu}\right)^{2r}$ 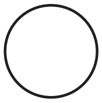 $= \left(\frac{|H|}{d_\mu}\right)^{2r} d_\mu$ (69)

The lemma can be proved by plugging this result into the expression for $\mathcal{I}$. □

We can now use this lemma to compute $\mathrm{Tr}(\tilde{\rho}^n)$, by identifying r with $n-1$ and H with $Z(k)$. Suppose k belongs to the conjugacy class C. Then $Z(k)$ is isomorphic to Z_C which is the centralizer of some arbitrary representative of C. We then find

$$\mathrm{Tr}(\tilde{\rho}^n) = |G|^{4L+6n-4} \sum_{(C,\mu)} (|C| d_\mu)^{4-2n} \,, \tag{70}$$

where $\mu \in \mathrm{Irrep}(Z_C)$, and we have used $|G| = |C||Z_C|$. In particular, $\mathrm{Tr}\,\tilde{\rho} = |G|^{4L+4}$, where we used $\sum_\mu d_\mu^2 = |Z_C|$. We can then obtain the Renyi entropy

$$S^{(n)}_{T_2\cup T_4} = S^{(n)}_{T_1\cup T_3} = \frac{1}{1-n}\log\left[\frac{\mathrm{Tr}(\tilde{\rho}^n)}{(\mathrm{Tr}\,\tilde{\rho})^n}\right] \tag{71}$$

$$= 4L\log|G| + \frac{1}{1-n}\log\left[\sum_{(C,\mu)}\left(\frac{d_{(C,\mu)}}{|G|}\right)^{4-2n}\right], \tag{72}$$

where $d_{(C,\mu)} := |C| d_\mu$. Recall again that we have assumed the four $T_i T_{i+1}$ interface circles to have the same length L. In general the $4L$ factor here should be replaced with the total interface length.

D. Mutual Information on a Torus

Using the previous results, we get

$$I^{(n)}(T_1, T_3) = \frac{1}{n-1}\log\left[\sum_{(C,\mu)}\left(\frac{d_{(C,\mu)}}{|G|}\right)^{4-2n}\right], \tag{73}$$

suggesting that $d_{(C,\mu)}$ are nothing but the anyon quantum dimensions.

[1] A. Y. Kitaev, Fault-tolerant quantum computation by anyons, Annals of Physics **303**, 2 (2003), arXiv:quant-ph/9707021 [quant-ph].

[2] S. X. Cui, D. Ding, X. Han, G. Penington, D. Ranard, B. C. Rayhaun, and Z. Shangnan, Kitaev's quantum double model as an error correcting code, Quantum **4**, 331 (2020), arXiv:1908.02829 [quant-ph].

[3] M. Levin and X.-G. Wen, Detecting Topological Order in a Ground State Wave Function, Phys. Rev. Lett. **96**, 110405 (2006), arXiv:cond-mat/0510613 [cond-mat.str-el].

[4] We note that after this edge duplication, the resulting state can be regarded as the ground state of the quantum double model on the extended lattice. The requirement that each pair of spins obtained from duplication have the same group element state is ensured by the holonomy free condition. Therefore, the process of edge duplication does not change the TO of the state.

[5] Y. Hu and Y. Wan, Entanglement entropy, quantum fluctuations, and thermal entropy in topological phases, Journal of High Energy Physics **2019**, 110 (2019), arXiv:1901.09033 [cond-mat.str-el].

[6] Y. Hu, Y. Wan, and Y.-S. Wu, Twisted quantum double model of topological phases in two dimensions, Phys. Rev. B **87**, 125114 (2013), arXiv:1211.3695 [cond-mat.str-el].

[7] Y. Hu, N. Geer, and Y.-S. Wu, Full dyon excitation spectrum in extended Levin-Wen models, Phys. Rev. B **97**, 195154 (2018), arXiv:1502.03433 [cond-mat.str-el].

[8] A. Kitaev and J. Preskill, Topological Entanglement Entropy, Phys. Rev. Lett. **96**, 110404 (2006), arXiv:hep-th/0510092 [hep-th].
